# The Road to a World Health Organization Global Strategy for Reducing the Harmful Use of Alcohol

**Published:** 2011

**Authors:** Maristela G. Monteiro

Harmful alcohol use and the related health effects are a global problem and therefore need to be addressed not only by individual nations but also on an international level. For example, the World Health Organization (WHO) noted that harmful alcohol use is the third leading risk factor for premature deaths and disabilities in the world, accounting for approximately 2.5 million deaths worldwide (corresponding to 3.8 percent of all deaths) in 2004 (WHO 2010). Moreover, harmful alcohol use was considered responsible for 4.5 percent of the global burden of disease as measured in disability-adjusted life-years lost in the same year. Given this scope of the impact, the WHO initiated a series of efforts that culminated in the development of a global strategy for reducing the harmful use of alcohol. This article reviews the alcohol-related activities of the WHO over the years and summarizes the central issues addressed by the global strategy.

## Historical Overview of WHO Activities Focusing on Harmful Alcohol Use

As early as 1979, the WHO initiated a program focusing on alcohol-related problems. This program assessed the impact of alcohol consumption in developing and developed societies and has coordinated dozens of projects and activities that have helped build the evidence, awareness, and support necessary for the development of a global alcohol strategy.

In 1997, the WHO also created the Global Information System on Alcohol and Health (GISAH) (http://apps.who.int/globalatlas/default.asp), which currently is hosted and maintained by the Centre for Addiction and Mental Health in Toronto, Canada. This information system is compiling the most reliable and updated information in the world on alcohol consumption and related harm by country. It includes and regularly updates data on recorded alcohol production, on alcohol consumption and related health effects based on national surveys and estimations of unrecorded consumption, and on national alcohol policies and interventions. The database has information from most countries around the world, although many gaps in the validity and reliability of the information remain.

In 1999, the WHO published its first *Global Status Report on Alcohol,* which relied on a combination of national and regional estimates, industry data, and data from the United Nations (UN) Food and Agriculture Organization and UN Statistical Office to generate estimates of adult per capita consumption for most countries (WHO 1999). Subsequently, three additional reports have been published, including the *Global Status Report: Alcohol and Young People* (WHO 2001), the *Global Status Report: Alcohol Policy* (WHO 2004*a*), and the *Global Status Report on Alcohol 2004* (WHO 2004*b*), and a new global report was published at the end of 2010.

A subsequent study conducted in 2000—the *WHO Comparative Assessment of Risk Factors for the Global Burden Disease—* demonstrated that alcohol consumption ranked as the fifth most important risk factor worldwide (Rehm et al. 2003; WHO 2002). Furthermore, alcohol was identified as the leading risk factor in developing countries with low mortality rates and the third-leading factor in developed countries. These findings clearly indicated a need for global action regarding alcohol’s harmful effects.

## The Global Strategy to Prevent Alcohol’s Harmful Effects

In 2005, the World Health Assembly (WHA) approved a resolution on public health problems caused by harmful use of alcohol (WHA 58.26), thus recognizing that alcohol has a worldwide impact and that strategies exist to reduce such an impact. This resolution led to intensified work on reviewing the evidence on alcohol policies through an expert committee meeting and report. In addition, among other initiatives, the information in the global alcohol database was updated, followed by new estimates of the burden imposed by alcohol on global health. However, when presented at the WHA in 2007, these additional data still were insufficient to reach a consensus on a global alcohol strategy. Instead, the WHA in 2008 recommended a worldwide consultation process aimed at developing a draft strategy to be presented in 2010 (WHA 61.4).

This consultation process began with a Web-based open consultation, followed by consultations with economic operators (i.e., alcohol producers and distributors, bar and restaurant associations, the hospitality industry, and others with an interest in the selling of alcohol), nongovernment organizations (NGOs), health professionals, UN Agencies, and intergovernmental organizations. Based on this input, the WHO Secretariat developed a discussion article that included the elements that could be considered for a global strategy. Between February and May 2009, this discussion article was presented at six regional consultations,^1^ with focal points from the Ministries of Health of the countries in those regions. At each consultation, the Secretariat presented and received suggestions on the options and collected best practices from the countries, with special emphasis on at-risk populations, young people, and those affected by the harmful drinking of others. Based on all of these consultations, in which more than 100 countries participated, a draft strategy was developed and sent to all member states for review and comments. An additional consultation with member States was held in Geneva, Switzerland, in October 2009, before the document was finalized and presented at the Executive Board meeting of the WHO in January 2010. The strategy was approved to be presented at the WHA in May 2010, where it finally was approved by consensus on May 21 (WHA 63.10).

The strategy calls on each nation to conduct its own internal assessment and develop a strategy and surveillance system to monitor progress in reducing alcohol’s harmful effects. Furthermore, each nation is urged to develop its own permanent coordinating entity (e.g., a national alcohol council) that should include senior representatives from all involved departments of government as well as representatives from civil society and relevant professional associations. Finally, each nation should identify clear and objective strategies that are tailored to local circumstances and which include measurable targets based on available evidence.

All efforts and interventions that are implemented based on this strategy both at the national and at the international levels should adhere to the following guiding principles (WHO 2010):

- Public policies and interventions to reduce alcohol problems should be guided by public health interests and should be based on clear public health goals and on the best-available evidence.
- Policies should be equitable and sensitive to national, religious, and cultural contexts.
- All involved parties have the responsibility to act in ways that do not undermine the implementation of public policies and interventions to prevent and reduce harmful use of alcohol.
- Public health should be given proper deference in relation to competing interests, and approaches that support that direction should be promoted.
- Protection of populations at high risk of alcohol-attributable harm and those exposed to the effects of harmful drinking by others should be an integral part of policies addressing the harmful use of alcohol.
- Individuals and families affected by the harmful use of alcohol should have access to affordable and effective prevention and care services.
- Children, teenagers, and adults who choose not to drink alcoholic beverages have the right to be supported in their nondrinking behavior and protected from pressure to drink.
- Public policies and interventions to prevent and reduce alcohol-related harm should encompass all alcoholic beverages and surrogate alcohol.

The strategy includes policy options and interventions for implementation at the national level that fall into 10 complementary areas (WHO 2010):

- Leadership, awareness and commitment—this area encompasses, for example, the development or strengthening of national and subnational strategies for reducing harmful alcohol use, coordination of national strategies with other relevant sectors, broad access to information and education on alcohol use and its consequences, and public awareness programs.
- Health services response—this area may include policy options and interventions such as increasing the capacity of health and social welfare systems for implementing prevention and treatment efforts and strengthening initiatives for screening and brief interventions for harmful drinking in a range of health care settings.
- Community action—this area encompasses, among others, efforts to strengthen the ability of local authorities to encourage and coordinate community action, to provide information about effective community-based interventions, and to mobilize communities to prevent sale and consumption of alcohol by underage drinkers.
- Drinking-and-driving policies and countermeasures—this area includes interventions such as setting upper limits for blood alcohol concentrations, conducting sobriety checks, and encouraging provision of alternative transport (e.g., public transport) until after the closing time of alcohol-serving establishments.
- Availability of alcohol—this area includes potential measures such as systems to regulate production and distribution of alcohol (e.g., by limiting the number of establishments or hours of retail sales), setting minimum legal drinking ages, or adopting policies to prevent the sale of alcohol to intoxicated people or underage drinkers.
- Marketing of alcoholic beverages—this area encompasses measures to regulate marketing of alcoholic beverages (e.g., by regulating the content, volume, and media of marketing efforts) and to monitor adherence to these regulations.
- Pricing policies—measures in this area may include an appropriate taxation system, setting minimum prices for alcoholic beverages, and banning or restricting price-based promotions of alcoholic beverages.
- Reducing the negative consequences of drinking and alcohol intoxication—this area may include such diverse measures as permitting sale of alcohol and large-scale events only in plastic containers to minimize violence, reducing the alcoholic strength of different beverage categories, and providing shelter or care for severely intoxicated people.
- Reducing the public health impact of illicit alcohol and informally produced alcohol—this area includes interventions to ensure quality control in the production and distribution of alcoholic beverages, track and trace illicit alcohol production, and issue relevant public warnings about health threats from informal or illicit alcohol.
- Monitoring and surveillance—activities within this area could include establishing an agency or organization to collect, analyze, and disseminate data on alcohol use and abuse or defining a set of indicators of harmful alcohol use and of the effectiveness of policies and interventions to prevent that harmful use that could be tracked over time.

The process of implementing and monitoring the strategy at the global and national levels will be defined by member states in consultation with the WHO. The first meeting of national counterparts for the global strategy was held in Geneva, Switzerland, in February 2011. Although the strategy is not binding as the Framework Convention on Tobacco Control,^2^ this global effort is an attempt to increase national responses to alcohol problems with greater visibility and accountability. Progress will be measured through regular updates of the global information systems and estimates of the global burden of disease from alcohol.

However, significant challenges remain in implementing the global strategy. The globalization of the alcohol industry has increased the power of a few producers (especially beer producers) that are able to influence policies at the country level and protect their commercial interests, which are in conflict with public health goals. Trade agreements also are a potential threat if they do not protect public health policies aimed at saving lives and protecting young people from marketing and alcohol promotion. Finally, the alcohol field does not have as strong an advocacy voice from civil society as does the tobacco field, giving a further advantage to an already powerful industry. Strong political activity at both the national and international level will be necessary to promote a public health approach to alcohol consumption and change the tide of alcohol-related disease and disability worldwide.

## Summary

This global strategy marks the first time that the need for a global effort to reduce a public health threat such as the harmful consequences of alcohol use has been recognized. By devising this strategy, the WHO has paved the way for international work on tackling alcohol problems at the global, regional, and national levels, so that a measurable reduction of the impact of alcohol consumption on global health can be achieved in a foreseeable future. This strategy represents an important progress in the fight against the harmful effects of alcohol by demonstrating a collective commitment by the WHA to increase the level of activity in this area; the next step will be to match this commitment with the appropriate resources.

## Footnotes

1 The WHO divides the world into six regions, including the Region of the Americas, the European Region, the South-East Asia Region, the Western Pacific Region, the African Region, and the East Mediterranean Region.

2 The WHO Framework Convention on Tobacco Control (WHO FCTC) is the first treaty negotiated under the auspices of the WHO. It was developed in response to the globalization of the tobacco epidemic and is an evidence-based treaty that reaffirms the right of all people to the highest standard of health. It became effective on February 27, 2005, and has become one of the most widely embraced treaties in UN history.
